# Anti-escaping of incident laser in rare-earth doped fluoride ceramics with glass forming layer

**DOI:** 10.1038/s41598-019-56902-0

**Published:** 2019-12-30

**Authors:** H. F. Shi, P. J. Lin, J. X. Yang, J. L. Yuan, E. Y. B. Pun, Y. Song, X. Zhao, H. Lin

**Affiliations:** 1 School of Textile and Material Engineering, Dalian Polytechnic University, Dalian, 116034 P.R. China; 2 Faculty of Maritime and Transportation, Ningbo University, Ningbo, 315832 P.R. China; 3 Department of Electronic Engineering and State Key Laboratory of Terahertz and Millimeter Waves, City University of Hong Kong, Tat Chee Avenue, Kowloon, Hong Kong P.R. China

**Keywords:** Optoelectronic devices and components, Fluorescence spectroscopy

## Abstract

Adaptive fluoride ceramic with glass forming layer (GC_ZBL_-Er) used in laser anti-escaping has been prepared by one-step synthesis, and the thickness of glass layer is identified as ~0.41 mm. Blue, green and red emissions of Er^3+^/Yb^3+^ codoped fluoride ceramic (C_ZBL_-Er) and glass layer (G_ZBL_-Er) have been investigated under ~980 nm laser pumping. With the forming of thin glass layer on ceramic surface, the absorption intensities on diffuse reflection of GC_ZBL_-Er at 974 nm and 1.53 μm increase by 48% and 53% than those of C_ZBL_-Er. Excited by a 979 nm laser, the presence of the glass layer increases the absolute absorption rate in spectral power from 75% in C_ZBL_-Er to 83% in GC_ZBL_-Er, which is consistent with the improvement in the absorbed photon number. In addition, the quantum yield of GC_ZBL_-Er complex is raised by 28.4% compared to the case of ceramic substrate by photon quantification. Intense absorption-conversion ability and efficient macroscopical anti-escaping effect confirm the superiority of ingenious structure in the fluoride ceramics with glass forming layer, which provides a new approach for developing the absorption-conversion materials of anti-NIR laser detection.

## Introduction

With the development of modern optical technology, the laser device has been widely employed in material processing, laser designator, medical diagnosis and other fields^1–3^. Nowadays, the laser beams applied in optical communication and laser ranging include the 1.54 μm erbium (Er^3+^) doped glass laser and 980 nm high-power semiconductor laser^4–17^. Detection laser devices operate by two principles, one of which is to irradiate the laser beam on the attack target directly, such as laser blinding equipment, and then the other is to emit laser to the target and further receive the reflected wave, including ranging and semi-active laser guidance^18–22^. As the generation of laser equipment makes laser a threat for human heathy, the development of laser protection receives sustained attention. Absorptive typed laser-protective materials have been accredited as excellent candidates due to their wide applicability and convenient preparation^23–27^. On this basis, it is necessary to take some measures to greatly attenuate the intensity of the laser beam to achieve laser anti-escaping, which makes it better for anti-NIR laser detecting.

Rare earth (RE) elements possess unfilled 4 f electron layer structure, which produces a variety of energy levels, determining that RE ions doped materials can absorb photons of different wavelengths^28–41^. Among multitudinous RE ions, the level structure of Er^3+^ is rich and uniform, and Er^3+^ has strong absorption capacity in the ~980 nm and ∼1.53 μm ranges commonly used in laser detection^42–50^. In addition, Yb^3+^ ion as sensitized ion can further absorb the NIR radiation efficiently and transfer the excitation energy to Er^3+^ via energy transfer processes, which can achieve large NIR laser absorption and efficient up-conversion emission, presenting a great potential in laser protection materials^51–59^. At present, the glass matrix typed laser protective material can overcome the shortcomings of the poor heat resistance, easy aging and low chemical resistance of the plastic matrix typed. However, the absorption ability of the general glass matrix typed material to laser is difficult to meet the application of the anti-NIR laser detection. Therefore, the exploration focusing on a new-type compound material with excellent absorption-conversion ability and high heat resistance potential becomes urgent in the future.

In this work, enhanced typed fluoride ceramic with glass forming layer has been prepared by one-step synthesis, and the laser stealth and interference can be realized based on the principle of RE^3+^ absorption, light conversion and energy transfer for NIR detecting laser. The complex reflection process between the glass-ceramic transition region and glass layer promotes the continuous consumption of incident laser, which obviously improves the absorption efficiency of the G_ZBL_-Er. Here, the statement “anti-escape” is used to describe the material good absorption effect for incident laser light. In particular, the laser signals detected and tracked at 980 nm and 1.53 μm are strongly absorbed and converted into other wavelength light radiations. These results confirm that the special structure of fluoride ceramic equipped with glass layer can enhance absorption-conversion efficiency and laser anti-escaping effect for incident NIR rader laser.

## Discussion

### Structure and morphology property

**Figure 1:**
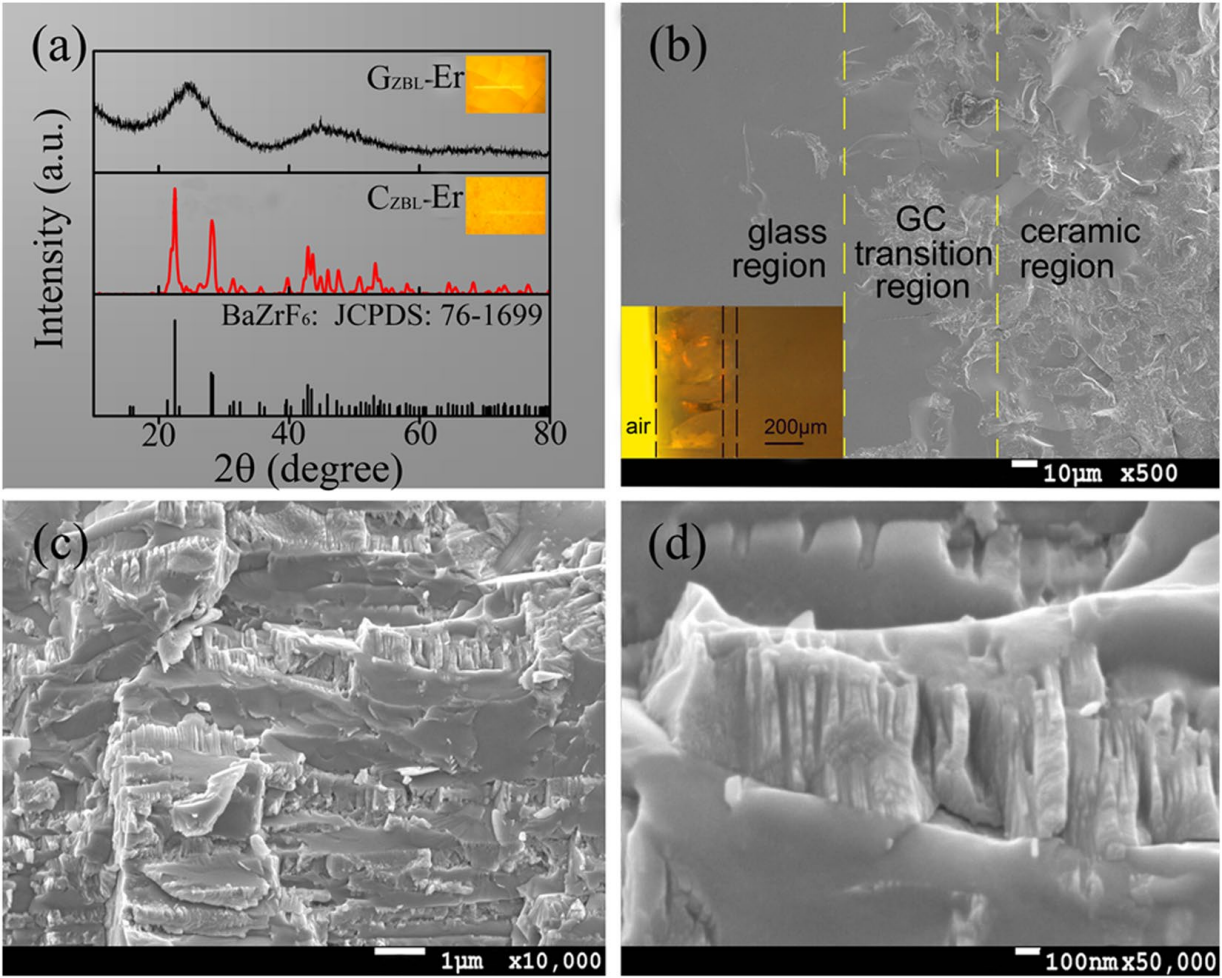
**(a)** XRD patterns of G_ZBL_-Er and C_ZBL_-Er powders. SEM images of **(b)** the glass-ceramic transition region and **(c,d)** the ceramic region of GC_ZBL_-Er. Inset: Corresponding photographys under fluorescence microscope.

To reveal absorption-conversion efficiency for NIR detecting laser, 1.0 wt% ErF_3_ and 2.0 wt% YbF_3_ as dopants are introduced into fluorozirconate matrix and denoted as GC_ZBL_-Er, and individual glass and ceramic phase are labeled as G_ZBL_-Er and C_ZBL_-Er, respectively. XRD pattern of as-synthesized G_ZBL_-Er powder exhibits a broad diffuse scattering at lower angles rather than the narrow diffraction peaks for crystal phase, and the amorphous nature of the G_ZBL_-Er glass layer is well identified, as exhibited in Fig. 1(a). In addition, the detected diffraction peaks of C_ZBL_-Er are in accordance well with the standard BaZrF_6_ (JCPDS 76–1699), and the derived cell parameters (a = 7.744 Å, b = 11.691 Å, c = 5.404 Å, α = β = γ = 90°) of C_ZBL_-Er are coincide with those of BaZrF_6_ phase (a = 7.681 Å, b = 11.357 Å, c = 5.511 Å, α = β = γ = 90°), indicating the formation of the pure BaZrF_6_ phase in upper layer of GC_ZBL_-Er composite. Meanwhile, a little difference in cell parameters is attributed to the crystalline environment variation and the lattice deformation with the introduction of Er^3+^ and Yb^3+^ ions to some extent.

The microstructure and morphology of as-synthesized GC_ZBL_-Er composite is explored, and the SEM image of the glass-ceramic transition region is displayed in Fig. 1(b). The interface between the glass and ceramic phase is obvious and uniform, and the thickness of the glass layer is measured to be ∼0.41 mm under the optical microscope. In addition, the grain size of the ceramics phase in Fig. 1(c,d) is identified to be 550 × 120 nm, and the crystalline phase of the rectangular structure with neat arrangement is further judged as the aggregation of several BaZrF_6_ crystallites.

**Figure 2:**
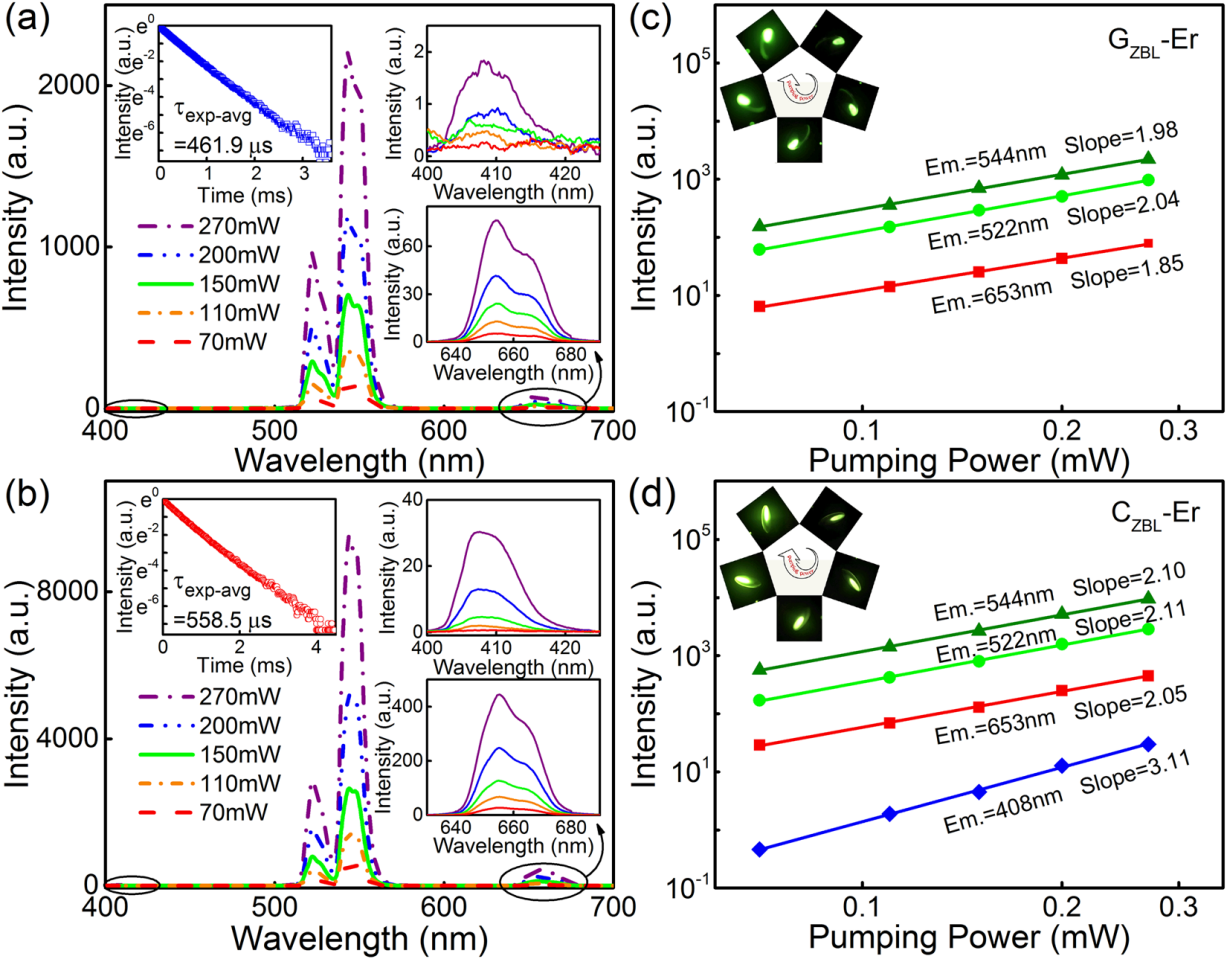
**(a,b)** Fluorescence emission spectra of G_ZBL_-Er and C_ZBL_-Er powders under 980 nm laser with various pumping powers. Inset: Corresponding fluorescence decay curves from the ^4^S_3/2_ level of Er^3+^ in G_ZBL_-Er and C_ZBL_-Er monitored at 544 nm under the 487 nm excitation. **(c,d)** Dependence of emission intensities on excitation powers in G_ZBL_-Er and C_ZBL_-Er. Inset: Correlative fluorescent photographs with the increment of doping concentration in the clockwise direction.

Under 980 nm laser excitation, the emission spectra of G_ZBL_-Er and C_ZBL_-Er powders with sundry pumping powers are depicted in Fig. 2(a,b), and four emission bands centered at 408, 522, 544 and 653 nm are attributed to f-f transitions ^2^H_9/2_ → ^4^I_15/2_, ^2^H_11/2_ → ^4^I_15/2_, ^4^S_3/2_ → ^4^I_15/2_ and ^4^F_9/2_ → ^4^I_15/2_, respectively. As the excitation power increases, the intensity of each peak increases exponentially, and the upconversion emission intensity *I*_lumin_ is proportional to the *n*th power of the 980 nm excitation intensity *I*_excit_, which can be simply expressed as ${I}_{{\rm{lumin}}}\propto {I}_{{\rm{excit}}}^{n}$, where *I*_lumin_ is fluorescence intensity, *I*_excit_ is excitation power and *n* is the number of 980 nm photons absorbed per visible photon emitted. In addition, the intense upconversion green and red emissions are confirmed to be two-photon absorption processes as indicated in Fig. 2(c,d), besides, rare 408 nm blue emission is identified as three-photon absorption process in the low phonon energy material.

In fluorozirconate glass system, the polyhedral network structure is formed mainly through Zr−F−Zr bridging, promoting the G_ZBL_-Er sample with low phonon energy ∼570 cm^−1^ ^60,61^. The characteristic can reduce the probability of non-radiative transition, which reflects intuitively in 544 nm emission of Er^3+^. The fluorescence decay curves of the ^4^S_3/2_ level for G_ZBL_-Er and C_ZBL_-Er layer monitored at 544 nm are exhibited in the inset of Fig. 2(a,b). In addition, the fluorescent lifetimes (τ_exp-avg_) of G_ZBL_-Er and C_ZBL_-Er are up to be 461.9 and 558.5 μs, respectively, which far exceed to 58 μs in Li_2_B_4_O_7_ glass^62^, 26 μs in SrF_2_ nanocrystals^63^, 333 μs in NaYF_4_^64^ and are close to 490 μs in oxyfluoride tellurite^65^, indicating that the low phonon-energy material contributes to photon releasing effectively.

### Enhanced absorption effect and principle analysis of GC_ZBL_-Er

**Figure 3:**
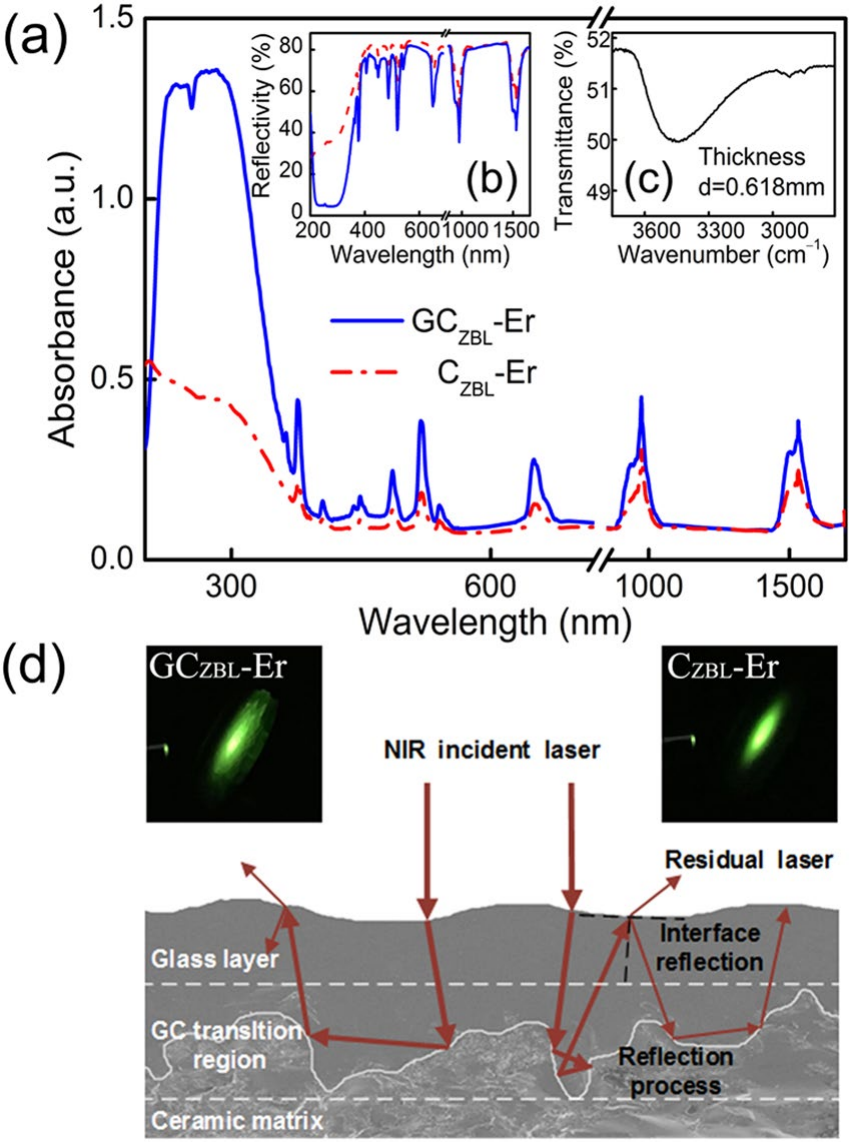
**(a)** Absorption spectra on diffuse reflection of C_ZBL_-Er and GC_ZBL_-Er samples. **(b)** Reflectivity spectra of C_ZBL_-Er and GC_ZBL_-Er. **(c)** FT-IR spectrum of glass layer. **(d)** Schematic diagram of GC_ZBL_-Er absorption process under NIR incident laser. Inset: Fluorescent photographs of C_ZBL_-Er and GC_ZBL_-Er under 980 nm laser pumping.

Although the optical transition capability of the glass fluorescent material to NIR laser are not as intense as crystal materials, when a glass forming layer is compounded on fluoride ceramics, the situation will be reversed. As exhibited in Fig. 3(a), the absorption intensities of GC_ZBL_-Er at 974 and 1532 nm are ∼1.48 and ∼1.53 times higher than those in C_ZBL_-Er, meanwhile. Correspondingly, the derived reflectance curve shown in Fig. 3(b) more clearly indicates that the reflectivity is reduced from 50% to 36% with the existence of thin glass layer. The reflected laser intensity including the Fresnel reflection is uniformly distributed and fully recorded in the integrating sphere, and the intensity radio of the reflected laser to incident laser can analyze the reflection ability of the material more macroscopically. Besides, the molar absorption coefficient *α*_OH_ can be used to evaluate the residual OH content in glass samples and is derived to be 0.91 cm^−1^ in 75TeO_2_–10ZnO–10Na_2_O–5GeO_2_ glasses^66^, while the value in this work glass is as low as 0.57 cm^−1^. The FT-IR spectrum of glass layer is shown in Fig. 3(c), and the low OH content is beneficial for anticipated photon emitting of this material. The apparent improvement can be attributed to the complex surface morphology of ceramic matrix and the intense dispersion effect of glass layer, and the schematic diagram of the absorption mechanism is shown in Fig. 3(d). When the detection laser is incident into the composite material GC_ZBL_-Er, it will be absorbed by the glass phase in the reflection process of the glass-ceramic transition region. Then the residual laser is re-reflected to the ceramic boundary owning to the specular effect of the glass layer, forming a multiple-cycle effect, which heightens the absorption ability effectively of GC_ZBL_-Er for NIR laser. In addition, the interface of the glass to ceramic region in schematic diagram is a rough outline, while the actual surface topography is more complicated in fact. So the absorption effect of NIR incident laser is greatly increased with the complexity of the reflection process in the GC_ZBL_-Er composite material. Just as the inset of Fig. 3(d), the facula area of GC_ZBL_-Er is bigger than C_ZBL_-Er and the luminous intensity is brighter at the same condition, showing that the GC_ZBL_-Er composite increases the absorption intensity effectively to the NIR incident laser with the forming of thin glass layer on ceramic surface. Besides, the surface of formed glass is quietly smooth and further can effectively solve the follow-up cleaning problems in application. These results indicate that the complex structure of GC_ZBL_-Er can be employed to enhance the absorption efficiency of ∼980 nm and ∼1.53 μm wavelengths, further exhibiting a laser anti-escaping effect.

### Photon quantification on absorption-conversion potential *of* C_ZBL_-Er and GC_ZBL_-Er

**Figure 4:**
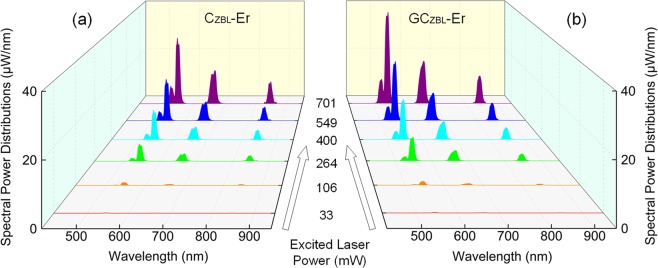
Net spectral power distributions of **(a)** C_ZBL_-Er and **(b)** GC_ZBL_-Er under the 979 nm laser excitation with different excitation powers.

**Figure 5:**
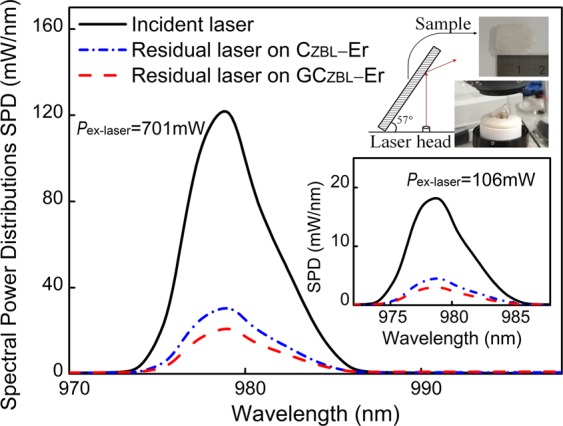
Spectral power distributions of the initial laser, the residual laser on C_ZBL_-Er and GC_ZBL_-Er under 701 mW and 106 mW laser powers in the integrating sphere. Inset: Location illustration of the sample measurement and the photograph of GC_ZBL_-Er under natural light.

In order to quantitatively characterize on absorption-conversion behavior of C_ZBL_-Er and GC_ZBL_-Er for laser beam, integrating sphere coupled with a CCD detector is applied to measure the absolute spectral parameters, which provides external quantum yield (QY) to evaluate luminescence and laser materials. Figure 4 presents the spectral power distributions as a function of 979 nm laser pumping power in C_ZBL_-Er and GC_ZBL_-Er samples, and the measured excitation powers are selected as 33, 106, 264, 400, 549 and 701 mW, respectively. Here, to ensure the laser fully diverged in integrating sphere, each sample is placed obliquely at the same angle and keeps a distance from the laser head. Besides, the tilt angle of the sample, the divergence angle of the laser and the distance of the laser head to the sample are measured to derive the area of the laser spot. Based on the above, the laser excitation power densities of the sample are further determined to be 16, 52, 129, 196, 269 and 344 mW/mm^2^, respectively. Taking the high-power 701 mW and low-power 106 mW incident laser as an example, the 980 nm incident laser, the residual lasers on the excited glass surface and ceramic surface are measured as shown in Fig. 5. Furthermore, the absorption ratio of C_ZBL_-Er to the incident laser is as high as 75.1% and 75.0% under 979 nm laser with 701 mW and 106 mW powers, respectively. Surprisingly, when the glass layer of GC_ZBL_-Er sample faces laser head, the absorption rates further rise to 83.4% and 82.9%, which is attributed to the intense dispersion of laser beam between glass and ceramic, proving that the special structure of composite glass layer is more suitable for the absorption of NIR laser light.

**Figure 6:**
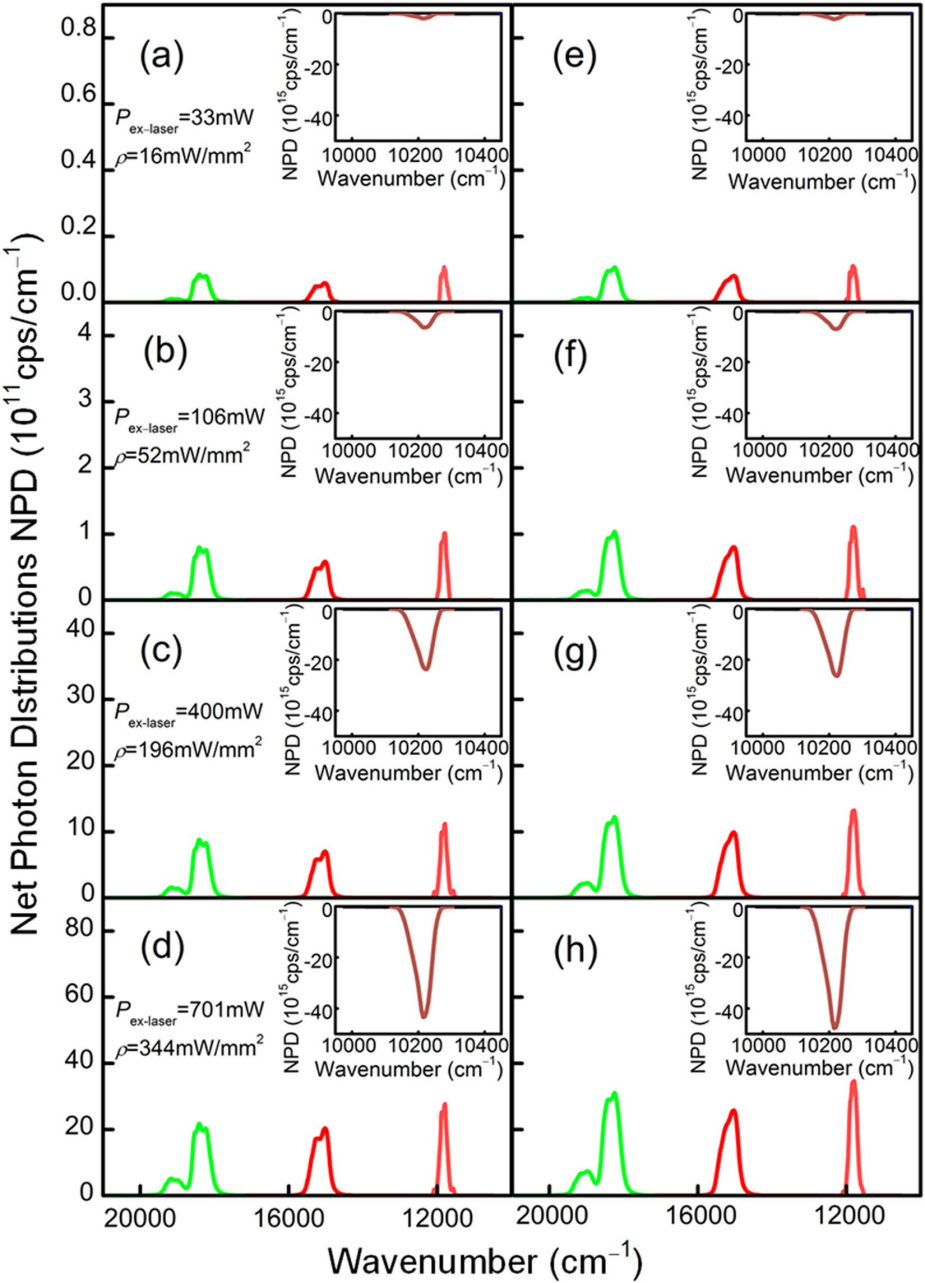
Net emission photon distributions in **(a–d)** C_ZBL_-Er and **(e–h)** GC_ZBL_-Er under the 979 nm laser excitation. Insets: details of related net absorption photon distributions of C_ZBL_-Er and GC_ZBL_-Er under the excitation of 979 nm laser in an integrating sphere.

**Figure 7:**
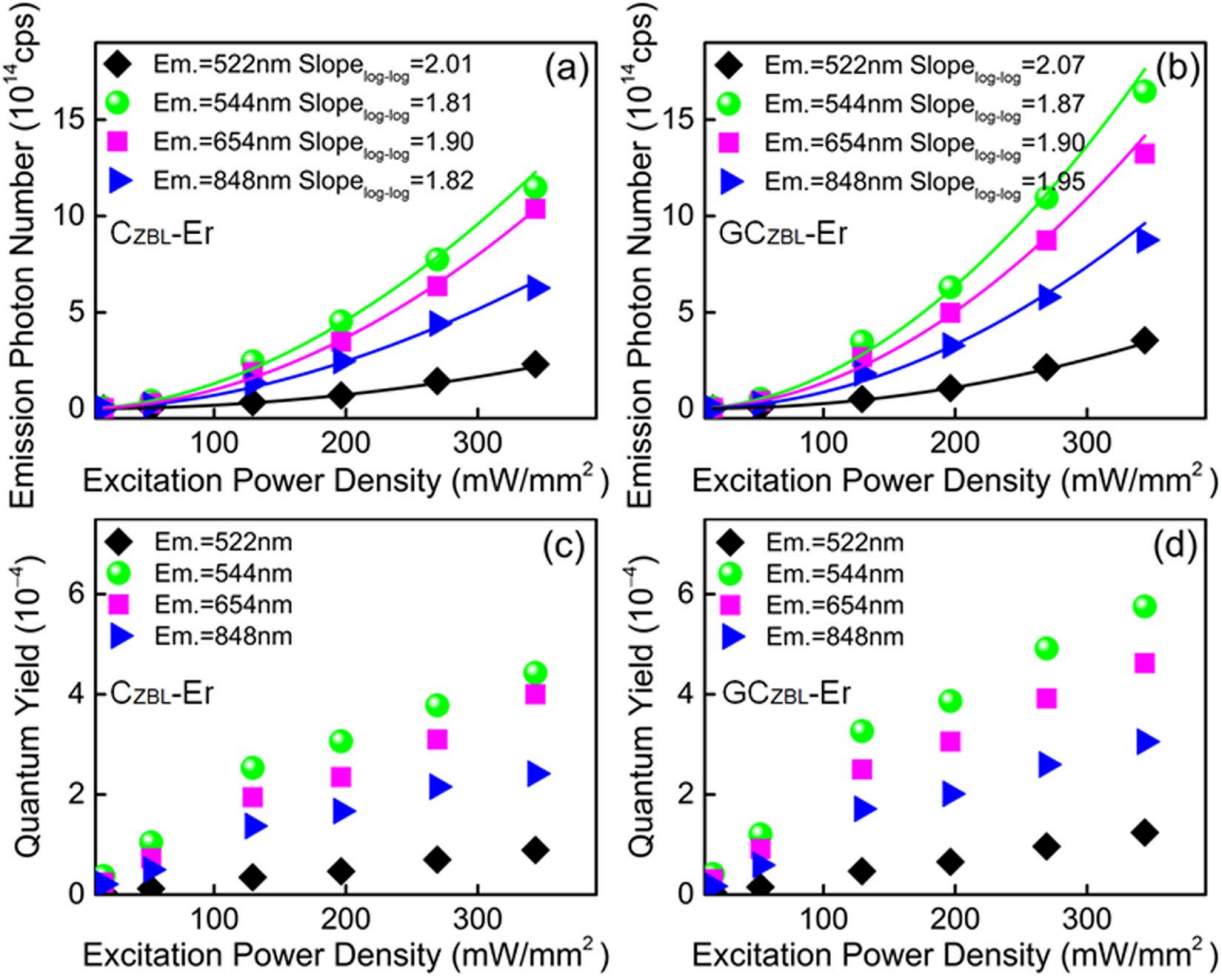
**(a,b)** Dependence of up-conversion emission photon numbers on excitation powers in C_ZBL_-Er and GC_ZBL_-Er. **(c,d)** Quantum yields of C_ZBL_-Er and GC_ZBL_-Er under the 979 nm laser with different excitation power density.

As a clear resolution of the photon number cumulative conversion effect, the photon number distribution can further elaborate the up-conversion emission law of the sample. The photon quantization is adopted to explain the enhanced absorption-conservation ability and anti-escaping effect of glass layer to NIR lasers. Based on the net spectral power distribution, the photon number distribution can be derived by $N(\nu )=\frac{{\lambda }^{3}}{hc}P(\lambda )$, where *λ* is the wavelength, *ν* is the wavenumber, *h* is the Planck constant, *c* is the vacuum light velocity, and *P*(*λ*) is spectral power distribution. The net absorption and emission photon distribution curves of C_ZBL_-Er and GC_ZBL_-Er are derived as presented in Fig. 6, and the integrated values are listed in Table 1. The green, red and NIR emissions at 522, 543, 665 and 848 nm are assigned to the ^2^H_11/2_ → ^4^I_15/2_, ^4^S_3/2_ → ^4^I_15/2_, ^4^F_9/2_ → ^4^I_15/2_ and ^4^S_3/2_ → ^4^I_13/2_ transitions of Er^3+^, respectively. In addition, the intense UC 848 nm emission is not easy to obtain, which provides more sufficient approaches for the conservation of incident laser. Since the ceramic substrate composites the glass layer, the emission intensity of GC_ZBL_-Er at wavenumber is stronger than that in C_ZBL_-Er material. When laser power density is selected to be 129 mW/mm^2^, the net emission photons of four emissions at 522, 543, 665 and 848 nm are as high as 2.31 × 10^14^, 11.49 × 10^14^, 10.38 × 10^14^ and 6.62 × 10^14^ cps of C_ZBL_-Er, respectively. Moreover, with the formation of the glass layer, emission photons further improve and reach to be 3.56 × 10^14^, 16.48 × 10^14^, 13.24 × 10^14^, 8.75 × 10^14^ cps in GC_ZBL_-Er, respectively. In addition, the enhanced percentage of total emitted photon number shows a trend of improving first and then decreasing slightly with the increase of laser pumping power. Figure 7(a,b) show the emission photon number of C_ZBL_-Er and GC_ZBL_-Er under the 979 nm laser with different excitation power density, where the rising tendency of the above four emission photons become dramatically severe, manifesting that the two-photon-excited luminescence has a positive dependency on the excitation power density.

**Table 1:** Absorption and emission photon numbers and enhanced percentage in the C_ZBL_-Er and GC_ZBL_-Er under the 979 nm laser excitation with different power densities.

Excitation power density (mW/mm^2^)	Sample	Net absorption photon number (10^16^cps)	Enhanced percentage (%)	Emission photon number (10^14^cps)					
				^2^H_11/2_ → ^4^I_15/2_	^4^S_3/2_ → ^4^I_15/2_	^4^F_9/2_ → ^4^I_15/2_	^4^S_3/2_ → ^4^I_13/2_	Total	Enhanced percentage (%)
16	C_ZBL_-Er	11.48	11.4	0.005	0.043	0.028	0.019	0.095	26.5
	GC_ZBL_-Er	12.79		0.006	0.053	0.0392	0.022	0.120	
52	C_ZBL_-Er	38.96	10.2	0.045	0.408	0.283	0.195	0.931	32.1
	GC_ZBL_-Er	42.93		0.066	0.517	0.392	0.255	1.230	
129	C_ZBL_-Er	97.05	9.9	0.338	2.458	1.890	1.332	6.018	41.0
	GC_ZBL_-Er	106.67		0.502	3.484	2.668	1.830	8.484	
196	C_ZBL_-Er	148.08	10.0	0.692	4.535	3.479	2.474	11.180	39.8
	GC_ZBL_-Er	162.87		1.067	6.298	4.979	3.282	15.626	
269	C_ZBL_-Er	205.03	8.6	1.435	7.750	6.356	4.423	19.964	38.4
	GC_ZBL_-Er	222.76		2.152	10.954	8.734	5.791	27.631	
344	C_ZBL_-Er	259.29	10.4	2.312	11.489	10.376	6.268	30.445	38.0
	GC_ZBL_-Er	286.36		3.556	16.479	13.241	8.751	42.027

The photoluminescence quantum yield (QY) is conducive to judge the luminous characters of optical materials, which provides a direct evaluation for the laser absorption-conversion efficiency. Thus the absolute fluorescence parameter of QY for C_ZBL_-Er and GC_ZBL_-Er are carried out based on $QY={N}_{{\rm{em}}}/{N}_{{\rm{abs}}}$, where the *N*_abs_ and *N*_em_ represent the net absorption photon number and net emission photon number The QYs for green, red and NIR UC emission of C_ZBL_-Er and GC_ZBL_-Er under 979 nm NIR laser with different pumping power densities are listed in Table 1 and illustrated in Fig. 7(c,d). As listed in Table 2, the total QYs of C_ZBL_-Er and GC_ZBL_-Er reach to be 0.86 × 10^−4^∼11.74 × 10^−4^ and 0.96 × 10^−4^∼14.67 × 10^−4^, respectively. The QY of the GC_ZBL_-Er up to 7.96 × 10^−4^ is solved under the excitation of 129 mW/mm^2^ power density, which is 28.4% more than that of C_ZBL_-Er. The QYs of green and red emissions from Er^3+^ and Ho^3+^ in different glass matrices are listed in Table 3. As can be seen from the data, the high quantum yield in GC_ZBL_-Er sample is over ten times higher than the values of BALMT glass, NMAG glass, BZYTLE glass and other oxide glasses^67–70^. However, the quantum yield in fluoride glass exceeds than that of GC_ZBL_-Er^67^, which is attributed to the superior absorption capacity of the composite with special structure. With the enhancing incident laser power densities, the number of photons emitted increases exponentially, and the quantum yield improves continuously, which indicates that both absorption and emission of C_ZBL_-Er and GC_ZBL_-Er for NIR laser are still not saturated. Taken together, these results manifests that the forming of glass layer on ceramic substrate not only improves the absorption for NIR laser by the multi-reflection process, but also greatly enhances the optical-conversion ability, which confirms the GC_ZBL_-Er complex processes a potential applied in anti-escaping of incident laser.

**Table 2:** Quantum yields of C_ZBL_-Er and GC_ZBL_-Er excited by the 979 nm laser.

Excitation power density (mW/mm^2^)	Sample	Quantum yield (10^−4^)					
		^2^H_11/2_ → ^4^I_15/2_	^4^S_3/2_ → ^4^I_15/2_	^4^F_9/2_ → ^4^I_15/2_	^4^S_3/2_ → ^4^I_13/2_	Total	Enhanced percentage (%)
16	C_ZBL_-Er	0.04	0.37	0.24	0.21	0.86	9.3
	GC_ZBL_-Er	0.05	0.41	0.31	0.17	0.94	
52	C_ZBL_-Er	0.12	1.05	0.73	0.50	2.40	14.6
	GC_ZBL_-Er	0.15	1.20	0.91	0.59	2.85	
129	C_ZBL_-Er	0.35	2.53	1.95	1.37	6.20	28.4
	GC_ZBL_-Er	0.47	3.27	2.50	1.72	7.96	
196	C_ZBL_-Er	0.47	3.06	2.35	1.67	7.55	27.3
	GC_ZBL_-Er	0.66	3.87	3.06	2.02	9.61	
269	C_ZBL_-Er	0.70	3.78	3.10	2.16	9.74	27.2
	GC_ZBL_-Er	0.96	4.91	3.92	2.60	12.39	
344	C_ZBL_-Er	0.89	4.43	4.00	2.42	11.74	25.0
	GC_ZBL_-Er	1.24	5.75	4.62	3.06	14.67

**Table 3:** Comparison of quantum yields for green and red emissions from Ho^3+^ and Er^3+^ in various glasses.

Glasses	QY of green emission (~550 nm)	QY of red emission (~650 nm)	Experimental method	References
Er^3+^ doped silicate glass	2.0 × 10^−7^	—	Relative method	^67^
Ho^3+^ doped NMAG	1.7 × 10^−6^	24.1 × 10^−6^	Absolute method	^68^
Er^3+^ doped NMAG	5.5 × 10^−6^	21.9 × 10^−6^	Absolute method	^68^
Er^3+^ doped BZYTLE (I)	2.3 × 10^−5^	1.4 × 10^−5^	Relative method	^69^
Er^3+^ doped BZYTLE (II)	3.3 × 10^−5^	0.7 × 10^−5^	Relative method	^69^
Ho^3+^ doped BALMT	6.3 × 10^−5^	8.9 × 10^−5^	Absolute method	^70^
Er^3+^ doped fluoride glasses	1.0 × 10^−3^	—	Relative method	^67^
Er^3+^ doped C_ZBL_	4.4 × 10^−4^	4.0 × 10^−4^	Absolute method	[This work]
Er^3+^ doped GC_ZBL_	5.8 × 10^−4^	4.6 × 10^−4^	Absolute method	[This work]

## Conclusion

Multi-photon-excited blue, green, red and NIR emissions have been quantified in Er^3+^/Yb^3+^ doped fluoride ceramic (C_ZBL_-Er) and glass layer (G_ZBL_-Er). The fluorescent lifetimes of G_ZBL_-Er and C_ZBL_-Er are up to be 461.9 and 558.5 μs, which indicates the fluorozirconate system can achieve effective photon releasing due to low maximum phonon energy. The absorption intensity of GC_ZBL_-Er at 974 and 1532 nm are determined to be ∼1.48 and ∼1.53 times higher than those in C_ZBL_-Er, and the absorption enhancement is attributed to the reflection of complex surface morphology on ceramic substrates and the diffusion absorption of glass layers. With the forming of thin glass film on ceramic surface, net absorption power and net absorption photon number of GC_ZBL_-Er exhibit an increase of ~10% by photon quantization in the integrating sphere. Corresponding the emission photon number and quantum yield enhance by 40% and 28%, respectively, and the higher photon release efficiency further implies the superiority of the special composite structure in light conversion. The high absorption-conversation efficiency attributed to the complex structure of transition layer confirms the macroscopical anti-escaping effect in GC_ZBL_-Er, which provides a reliable approach for anti-NIR laser detection.

## Methods

### Prototype design and fabrication of C_ZBL_-Er and GC_ZBL_-Er

The fluoride ceramic-based composite glass layers were prepared based on the molar host composition of 60ZrF_4_−30BaF_2_−10LaF_3_ (ZBL) via the melt-quench method in reducing atmosphere. In addition, 1.0 wt% ErF_3_ and 2.0 wt% YbF_3_ as dopants were introduced into ZBL matrix and denoted as GC_ZBL_-Er, and the individual glass phase and ceramic phase were labeled as G_ZBL_-Er and C_ZBL_-Er, respectively. The high-purity fluoride raw materials were melted at 900 °C for 5 min in a platinum crucible, and then the molten glasses were poured into a metal mold in a dry air atmosphere. Here, the lower liquid contacting with aluminum plate rapidly formed an ultrathin glass layer owing to the process of efficient heat conduction, where the metal mold quickly was taken away a lot of heat. Correspondingly, the upper liquid itself provided the energy needed for glass crystallization, which greatly promoted the formation of crystals and the adhesion of glass layers. Subsequently, all samples were annealed at 260 °C for 2 h, and then cooled down slowly to room temperature inside the furnace. For optical measurements, the annealed samples were sliced into pieces and polished into pieces with parallel sides.

### Measurement and characterization

The amorphous nature of G_ZBL_-Er and the crystal structure of C_ZBL_-Er were identified utilizing a Shimadzu XRD-7000 diffractometer with Cu-Kα radiation (*λ* = 1.5406 Å) operated at 40 kV and 30 mA. The morphological behaviors for the section of GC_ZBL_-Er were observed by a field-emission scanning electron microscope (SEM instrument, JEOL JSM-7800F). The transmittance spectra of glass were recorded by a Perkin-Elmer FTIR/NIR Spectrometer (FTIR). The thickness of the glass layer of the GC_ZBL_-Er was measured by a fluorescence microscope (Imaging system CK-500). Visible fluorescence spectra and fluorescence decay curves were determined by a Hitachi F-7000 fluorescence spectrophotometer equipped with an R928 photomultiplier tube (PMT) as a detector and a commercial Xe-lamp as an excitation source. Diffuse reflectance spectra of samples were recorded by Shimadzu corporation UV3600 spectrophotometer, and the NIR emission spectra were implemented under the 980 nm laser by an Ocean Optics NIR-Quest 2250 fiber spectrometer.

The absolute spectral parameters of C_ZBL_-Er and GC_ZBL_-Er samples were measured in an integrating sphere of 3.3 inch inner diameter (Labsphere), which was connected to an exciting 979 nm NIR laser source and a QE65000 CCD detector (Ocean Optics) with 400μm-core and 600μm-core optical fibers, respectively. A standard halogen lamp (Labsphere, SCL-050) was adopted to calibrate this measurement system, and the spectral power distributions were obtained through fitting the factory data based on the black body radiation law.

## Data Availability

All data regarding the work presented here is available upon reasonable request to the corresponding author.
